# Beyond MRI surveillance in radiologically isolated syndrome: the emerging role of VR/AR neurotechnology for subclinical progression

**DOI:** 10.1097/MS9.0000000000004888

**Published:** 2026-04-13

**Authors:** Aneeq Mahmood Khan, Liaqat Kareem, Mahnoor Fatima

**Affiliations:** a Department of Medicine, Sargodha Medical College, Sargodha, Pakistan; b Department of Medicine, Air university, Islamabad, Pakistan; c Department of Medicine, Shaheed Ziaur Rahman Medical College, Rajshahi Medical University, Bogura Bangladesh

*To the Editor,*

Radiologically isolated syndrome (RIS) is an asymptomatic condition characterized by magnetic resonance imaging (MRI) lesions that meet the criteria for the dissemination in space of multiple sclerosis (MS), and it carries a significant risk of progression to symptomatic disease, including the more advanced forms. The presence of lesions in the spinal cord and infratentorial region greatly increases this risk; in a prospective study conducted on a cohort of 747 individuals, Groups 1 and 2 (one or two 2017 DIS criteria) with spinal cord lesions and CSF oligoclonal bands had a 38% 5-year clinical event risk, which was similar to 2009-RIS (38.7%). These lesions are indicative of direct transition to primary progressive MS; hence, the essential need for subclinical monitoring tools that are more efficient than just conducting periodic MRI[1].

Subclinical progression very rarely presents as cognitive mimicking of early progressive MS, with processing speed, executive function, and memory impairment being detectable in RIS preceding the appearance of overt symptoms. In relapsing-remitting MS (RRMS) with very mild disability (Expanded Disability Status Scale ≤2.5), half of the patients show memory and executive cognitive deficits, which are similar to the patterns in RIS and hint at the conversion. Progressive MS groups demonstrate a 52.4% prevalence of cognitive impairments at the end of 4 years, mainly affecting visual memory and processing speed, which does not depend on RRMS courses. Such changes that cannot be detected go undetected by the standard assessments; thus, intervention in high-risk RIS patients is delayed^[^2,3^]^.

Even though there is a thorough comprehension of the radiologic risk, the clinical instruments for revealing subclinical neurodegenerative change, especially minor cognitive decline, have not yet been invented. Cognitive dysfunction usually occurs only after considerable neuroaxonal loss, and it is not adequately detected by standard neurological examinations or periodic MRI alone. Digital technologies such as virtual reality (VR) and augmented reality (AR) are, by their nature, interactive and measurable platforms that allow for the repeated assessment of complex sensorimotor and cognitive performance. To date, most of the clinical studies of VR in MS have been related to rehabilitation, but they also point to significant cognitive gains in areas such as attention and executive function in different neurological groups, thus showing that VR metrics are sensitive enough to pick up functional change[4].

VR/AR neurotechnology provides biofeedback for early diagnosis of faint neurochanges through immersive tasks that monitor eye movements, response time, and neural activity patterns. Full immersion in VR rehabilitation is good for cognitive, short-term memory, and depression risk in MS and cognitive impairments, alongside semi-immersive formats being helpful for balance and attention. Newest studies reveal that VR training not only increases the non-trained memory, speed of processing, and motor function in patients with MS but also utilizes multisensory feedback for heightened detection of early decline. Wearables that are integrated with the system are able to provide constant biofeedback that reflects the subclinical shifts that are normally overlooked by conventional neuropsychometrics[5].

The combination of neurotechnology metrics with artificial intelligence (AI)-assisted analytics could lead to further optimization of the detection of subclinical progression. High-dimensional VR/AR performance data might be used to train AI models that can discern the patterns of decline or motor-cognitive dissociation, which are, in fact, before the occurrence of structural lesion accumulation on MRI. These kinds of multimodal approaches have been successful in working with mild cognitive impairment, where VR-derived metrics together with imaging biomarkers have greatly increased the classification accuracy compared to either modality alone[6]. We propose to have AI integrated with VR/AR platforms to make RIS monitoring personalized, where the analysis of biofeedback data will lead to anomaly detection and predictive alerts based on the progression thresholds. This proposal expands on the already established sensitivity of VR in neurodegenerative monitoring, which can possibly stratify spinal/infratentorial RIS for the initiation of targeted disease-modifying therapy. A pilot integration could take advantage of the existing RIS cohorts, thus providing cost-effective, home-based tools that are superior to MRI frequency^[^1,5^]^.

The possible effects on clinical care are significant. A VR/AR monitoring platform powered by neurotechnology could provide personalized, always-on evaluation in high-risk patients with RIS, and in that way, it would be the first to detect even minute neurofunctional change, support timely intervention, and provide risk stratification that is dynamic and personalized, adapting to individual drivers. If this system is equipped with instant AI warnings for performance ankle kicking beyond normal limits, doctors could be notified before the disease gets worse, thus changing the whole issue from being a reactive diagnosis to predictive precision monitoring. Such devices might also be considered as the main indicators of success in the clinical trials with early neuroprotective or immunomodulatory treatments targeting RIS populations.

In summary, integrating VR/AR neurotechnology with embedded AI analytics represents a significant advancement in monitoring subclinical worsening in RIS and preventing the progression of MS. We recommend that researchers conduct well-designed longitudinal studies to compare the performance of VR/AR biomarker technology with the gold standard of radiologic and clinical outcomes. This approach may support the inclusion of VR/AR in early MS management programs. This letter is in line with the TITAN Guidelines on the need for transparency in AI use in healthcare[7].

## Data Availability

Not applicable.
